# Design, planning and implementation lessons learnt from a surgical multi-centre randomised controlled trial

**DOI:** 10.1186/s13063-019-3649-0

**Published:** 2019-11-01

**Authors:** Katie Biggs, Daniel Hind, Mike Bradburn, Lizzie Swaby, Steve Brown

**Affiliations:** 1 Sheffield Clinical Trials Research Unit (CTRU), University of Sheffield, Regent Court, Sheffield, S1 4DA UK; 2 Department of Surgery, Sheffield Teaching Hospitals NHS Foundation Trust, Northern General Hospital, Herries Road, Sheffield, S5 7AU UK

## Abstract

**Background:**

Increasingly, pragmatic randomised controlled trials are being used to evaluate surgical interventions, although they present particular difficulties in regards to recruitment and retention.

**Methods:**

Procedures and processes related to implementation of a multi-centre pragmatic surgical randomised controlled trial are discussed. In this surgical trial, forecasting of consent rates based on similar trials and micro-costing of study activities with research partners were undertaken and a video was produced targeting recruiting staff with the aim of aiding recruitment. The baseline assessments were reviewed to ensure the timing did not impact on the outcome. Attrition due to procedure waiting time was monitored and data were triangulated for the primary outcome to ensure adequate follow-up data.

**Results:**

Forecasting and costing ensured that the recruitment window was of adequate length and adequate resource was available for study procedures at multiple clinics in each hospital. Recruiting staff found the recruitment video useful. The comparison of patient-reported data collected prior to randomisation and prior to treatment provided confidence in the baseline data. Knowledge of participant dropout due to delays in treatment meant we were able to increase the recruitment target in a timely fashion, and along with the triangulation of data sources, this ensured adequate follow-up of randomised participants.

**Conclusions:**

This paper provides a range of evidence-based and experience-based approaches which, collectively, resulted in meeting our study objectives and from which lessons may be transferable.

**Trial registration:**

ISRCTN, ISRCTN41394716. Registered on 10 May 2012.

UKCRN Study ID: 12486.

## Background

Randomised controlled trials (RCTs) are becoming more widely used to assess surgical interventions despite historical resistance [1–3]. However, a review across surgical specialities showed over 20% (81/395) of trials are prematurely discontinued [4], with poor recruitment being the principal reason (36/81); these discontinued trials recruited 15,626 participants. The discontinuation of trials results in considerable wasted investment and at best a less precise answer to the research question [5–7]. Poor recruitment and retention can lead to withdrawal of funding to complete the trial, which has financial and ethical implications [5, 6, 8].

McCulloch and colleagues [1] identified different classes of surgical trials with different levels of risk in terms of successful project completion: type 1 trials compare medical management in surgery; type 2 compare surgical techniques; and type 3 compare surgical and non-surgical treatments. Type 3 trials are particularly prone to a lack of clinician and patient equipoise [1]. Recruitment to trials with treatments of differing intensity are often poor [1, 9], with randomized-to-screened ratios of 1:16 documented [10].

The most common patient-reported reasons for non-entry into surgical RCTs are treatment preference or dislike of randomisation [11, 12] and, where treatments are markedly different, there is increased likelihood of patients or clinicians declaring a preference. In addition, recruiting clinicians often struggle to explain concepts such as randomisation and equipoise [13–15] and with the amount and clarity of information provided during the consent process [8]. There have been several articles looking at strategies to improve recruitment and retention in trials [5, 8, 13, 16–20] and evidence for successful interventions is limited. Qualitative work alongside surgical trials can identify particular issues around recruitment and can train staff to address the absence of equipoise and other issues [13].

The time waited from consent to surgery is a common reason for attrition [21, 22] and type 3 trials may lead to a greater difference in waiting times between treatment groups than types 1 or 2. With increasing waiting times a problem for some health systems [23], this should be a consideration in surgical trial design. Baseline measures such as health-related quality of life (HRQoL) often change over time, meaning long waiting times between consent and surgery are a potential source of bias. If baseline measures are taken on the day of surgery there is a possibility that the measures could be affected by the knowledge of the treatment allocation [24]. The clinically intuitive timing for follow-up measures is a timepoint relative to the day of surgery, whereas the scientifically desirable timing is a timepoint relative to the day of randomisation, although there is some evidence that this makes little difference to the reported outcomes [25].

Costing the resource required to deliver RCTs is an important factor in their success. Published workload models for organisations tend to use accrual data, acuity, or a points scale to estimate the research nurses and/or clinical trial administrator/co-ordinator resource needed to implement an RCT [6–11]. There is no consensus on which model best evaluates workload in clinical research infrastructure [12]. Systems that reimburse research infrastructure based on accrual data focus on the target accrual compared to the number of whole-time equivalents (WTEs), often without accounting for screen failures, query resolution, long-term follow up, participant attrition, or the complexity of the research protocol. They are criticised for over-simplicity and implicated in staff burnout and poor quality standards [12, 13]. This paper presents lessons from the Haemorrhoidal artery ligation (HAL) versus rubber band ligation (RBL) for haemorrhoids (HubBLe) trial [26–28], a type 3, multi-centre, surgical RCT, to support the implementation of future studies.

## Methods

### Summary of trial design and procedures

The aim of the HubBLe trial [26–28] was to establish the clinical effectiveness and cost effectiveness of HAL compared with RBL in the treatment of people with symptomatic second or third degree haemorrhoids. Both treatments are recommended for the treatment of haemorrhoids [29–32]. The trial was a pragmatic, multi-centre, parallel group RCT involving 18 National Health Service (NHS) hospitals in England and Scotland. After consent, participants were individually randomised to HAL or RBL in equal proportions at all centres using a web-based randomisation system. Participants were followed up at 1 day, 7 days and 21 days, 6 weeks, and 12 months post-procedure. Full details of the trial methods can be found elsewhere [26–28]; here, we focus on methods aimed at improving participant recruitment and retention to achieve a valid data set.

### Methods adopted to meet the recruitment target

#### Recruitment procedures

Eligibility criteria were broad in order to assure a large pool of patients from which to recruit, whilst ensuring patients were suitable for both procedures. HubBLe can be considered a type 3 trial in which medical management is compared with a surgical intervention [1, 9]: HAL was a procedure undertaken in theatre under general anaesthetic; RBL is a less intensive intervention, typically undertaken as an outpatient procedure, and is often delivered by non-surgeons. A key reason for under-recruitment in RCTs is over-optimism at the trial planning stage regarding how many people who are offered participation in the trial will consent and randomise [33–36].

In particular investigators often do not forecast based on a “reference class” of consent rates observed in previous similar trials [34, 37]. Prospect theory predicts that we are over-optimistic in our judgements, because we are overconfident and unaware or ignorant of existing data on similar projects (the “reference class”) [38–40]. For HubBLe, we made the following evidence-based assumptions about the recruitment activity based on a reference class of previous similar studies:

1. Many patients had to be screened for one patient to consent

For type 3 surgical trials, conversion rates rarely exceed 1 patient consented for every 5 screened and rates as low as one randomised for every 16 screened [9, 10]. We therefore anticipated that 12 patients will decline randomisation for each one who consents, a screening-to-randomisation ratio of 13:1.

2. Time spent per patient screened

Every patient screened would cost a research nurse 3 h in terms of liaison with the clinical team to ensure potentially eligible candidates were flagged; posting information about the study in advance of screening visits; time taken to get to screening visits in clinics; screening, information giving and discussion of equipoise issues; and consent and randomisation where required. For every patient recruited we requested costs for 38.5 h recruitment work. Assuming the conversion rate of 1 patient randomised in every 13 screened, recruiting 39 patients at a centre would require 1500 h (roughly 0.7 WTE over 1 year at each centre).

3. Coverage and rationalisation

There were an estimated two eligible patients available per clinic. With an estimate of four surgeons involved at every centre and an estimate of four clinics per centre per week, we recognised the challenge for recruiting research nurses to be available at all clinics with potentially eligible patients (coverage). Given the anticipated screening-randomised-ratio, it was imperative that as many potentially eligible patients were screened as possible. Research nurses worked with clinical teams to corral potentially eligible patients into particular clinics, especially where multiple surgeons share a waiting room. The research nurse could then use their time more efficiently with a view to minimising the number of unscreened patients (rationalisation).

#### Attribution and reimbursement of costs

The UK Government Department of Health’s system for attributing costs in NHS research and development (R&D) [41] means that resource for recruitment activity cannot be costed into grant applications. A case for supplementary funding for work relating to recruitment, “services support costs”, has to be made to a National Institute for Health Research (NIHR) Local Research Network (LRN) lead in the chief investigator’s locality. Once agreed, LRNs in other regions are expected to match the funding. The system has been the subject of criticism by researchers and delays associated with agreeing the allocation of costs have been documented [42–46]. To avoid such delays, we entered into discussion with the LRN, which began prior to the start of the study. The breakdown of research nurse costs for HubBLe are presented in Table 1; we ensured that the research nurse resource accounted for screen failures, participant attrition, data collection, data entry, query resolution, and the complexity of the research protocol.

**Table 1:** Costing of research nurse time per centre

Activity	Cost
Year 1 (recruitment and follow-up):	
Research nurse to screen and recruit patients (recruitment activity).	Research costs (research acitivty): 0.3 whole time equivalent (WTE) of a research nurse for the 12 month recruitment period for research activity.
Undertake data collection for the research, data entry, monitoring and meeting attendance (research activity).	Service support costs (recruitment activity): 0.7 WTE - We looked the LRNs to make up a full time post (based on our assumption about recruitment activity).
Year 2 (follow-up only):	
12 month follow-up (a half hour telephone interview plus data entry) and closeout visit, plus support for any monitoring and audit activity required.	Research costs: £300 per participant recruited

#### Recruitment video

In addition to ensuring sufficient recruitment capacity for the trial, the team developed a recruitment video [47] based on the ProtecT trial team’s work on the explanation of randomisation and equipoise [13]. We interviewed the local ProtecT trial team, two research nurses and a consultant involved in recruitment, about their recruitment experiences and narrated the film to highlight the general principles of equipoise and randomisation and how this specifically related to HubBLe. A recent systematic review of training interventions for trial recruiters [8] identified six trials [48–53] that had employed a video as part of a face-to-face workshop for that purpose, but there do not seem to be any training programmes solely using videos to aid recruiters. This was seen to be a low-cost method that could be referred to by recruiters as many times as they wished.

### Monitoring of waiting times

The duration between randomisation and treatment was monitored in the trial as we knew that there can be significant issues with waiting times for non-urgent surgeries, and that this could affect dropout rates and the intention-to-treat analysis. Whilst RBL is a simple procedure, which is often done on the day of randomisation, HAL is more intensive, is performed under general anaesthetic and requires a theatre slot to be booked. These conditions created the potential for differential participant attrition, a potential source of bias in our analysis [54, 55]. During the trial one of the centres stopped completing non-urgent surgery, which included our procedures. This has been shown to be a continuing issue for the NHS with one Clinical Commissioning Group (CCG) suspending non-urgent surgery to make financial savings prior to the end of the financial year in 2017 [56, 57]. Due to the long waiting times, for the HAL procedure in particular, and the cancellation of non-urgent procedures at one site, the dropout rate prior to the procedure was higher than anticipated. To account for this observed attrition the recruitment target was increased to 370, during the study, in order to achieve the sample of 350 treated, followed up and analysed participants.

### Changes in baseline health state post-randomisation, pre-surgery

Three months into recruitment the baseline data collection was changed to the day of procedure, rather than at randomisation. This was because there was substantial between-site variation in surgical wait times, and a difference in wait times for the two treatments, meaning that scores for change in patient-reported outcome at follow up may have reflected time periods substantially greater than intended, especially in the HAL arm. The risk of bias introduced by anchoring follow up to the time of surgery, rather than the point of randomisation, is theoretical and not supported by empirical evidence [25].

Six months after this change a Data Management and Ethics Committee (DMEC) member suggested that patient-reported outcomes can be affected by the knowledge of their allocation. Since baseline data collection took place on the day of surgery, most patients already knew their allocation by this point; the concern was that perceived pain and HRQoL may differ between the groups due to expectation bias at this time [24], even though no procedure had yet taken place. The trial statistician reported that the early data did indeed support this hypothesis, in particular with higher self-reported symptoms in the HAL arm. As a result of this, we added a questionnaire to be completed before randomisation as well as at baseline on the day of surgery if the two were more than 1 week apart.

### Methods to ensure a valid primary outcome data set

#### Three sources of primary data collection

Sometimes, in assessing an outcome, using only one data source may be unreliable and data source triangulation is necessary [58]. Our primary objective was to compare patient-reported symptom recurrence at 12 months following the procedure. Recurrence was defined using a simple dichotomous outcome derived from a previously published systematic review [59]. Patients were asked “At the moment, do you feel your symptoms are: cured or improved compared with before starting treatment; or unchanged or worse compared with before starting treatment.” We also asked patients whether (and which) procedures they had undergone for their haemorrhoids, further to trial treatment, since symptoms may only have resolved as a result of further intervention, and supplemented this with treatments as determined from their hospital notes and general practitioner (GP) in order to minimise attrition and recall bias. Finally, we reviewed adverse events and hospitalisations to identify participants that had ongoing symptoms consistent with persistent or recurrent haemorrhoids (e.g. persistent bleeding) that had not been treated.

## Results

### Recruitment

**Fig. 1:**
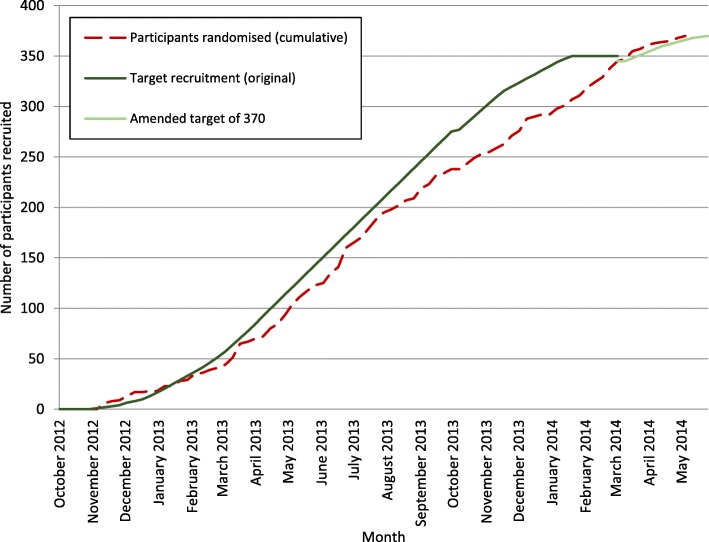
Participant recruitment graph. Reproduced with permission from Brown et al. 2016 [27]

Recruitment took place from 9 September 2012 to 6 May 2014, with follow up completed on 28 August 2015. The target and actual recruitment, including the increase in the recruitment target is shown in Fig. 1. There were 372 participants randomly assigned to receive RBL or HAL; 187 patients were allocated to receive RBL and 185 were allocated to receive HAL. Two of these participants (both allocated to RBL) were removed from the trial completely as they were ineligible at the time of consent, meaning a total of 370 participants were entered into the trial. An important observation is that less than one quarter of the sites (study sites 1, 2, 6, and 9) account for two thirds of the participants (251/372), while half of sites contributed one sixth of randomised participants.

Our early funding discussions with sites reduced delays in site set-up prior to the start of recruitment, and the lead site even started 1 month earlier than anticipated. Where sites agreed to our proposal, a full-time research nurse was dedicated to HubBLe during the recruitment period. Sites that exceeded their target recruitment (1, 2, 6 and 9) had a named research nurse responsible for the trial, as did sites 4, 7, and 8, though they did not recruit to target. An informal observation was that at sites where research nurses had less time to work on the trial, recruitment and the non-recruited data collection were generally poorer.

Of the 969 patients screened, 198 were not eligible (including the 2 patients that were withdrawn); the majority of these patients were not approached as clinical note review identified the exclusion criteria. The approximate randomised-to-screened ratio in the trial was 5:13; we therefore needed to formally screen (approximately) 13 people for every 5 randomised. This may underestimate the number of individuals screened, as the recording of data from non-recruited patients can be poor in clinical trials as the focus is on recruited participants.

Of the 401 eligible screened patients that were not recruited, 109 of these were not approached and 292 were invited to the trial but refused to consent; reasons for non-consent are shown in Table 2. Most patients who refused to consent did not want to be randomised due to their preference for a particular treatment (251/401, 62%).

**Table 2:** Reasons for non-enrolment to the trial

Reason	Frequency
Not eligible	**198**
Patient not approached	**109**
Clinical decision	41
Patient did not attend appointment/uncontactable	26
Unknown	42
Patient approached	**292**
Patient preference	251
Patient preference for RBL	128
Patient preference for HAL	70
Patient did not want any intervention or treatment	39
Patient preference for other surgery	5
Patient preference for immediate treatment	3
Patient preference related to general anaesthetic	6
Patient unsure or declined (no further reason given)	29
Other reason	12
Total	**599**

Adapted with permission from Brown et al. 2016 [27]. HAL haemorrhoidal artery ligation, RBL rubber band ligation

Bold text represents the higher-level reason for non-recruitment, with the detailed breakdown provided by the non-bold text

Bold text represents the higher-level reason for non-recruitment, with the detailed breakdown provided by the non-bold text

#### Video feedback

Although we did not assess the impact of the video on recruitment in any formal or structured sense, recruiting staff fed back that thinking about equipoise was very helpful, and that they found it easier to describe the two treatments after watching the video. Particular key points that were highlighted as helpful from the video were that it expressed the uncertainty of the effectiveness of each treatment; gave a similar amount of time discussing each treatment arm and avoid loaded statements that may communicate an unconscious bias for one treatment over another; and it provided the opportunity to check the patient’s understanding as you go.

### Withdrawals and waiting times

Overall, 35 participants withdrew from the trial, with 24 withdrawing from the HAL group and 11 from the RBL group; reasons for withdrawal are provided in Table 3. Only 3 participants withdrew after receiving treatment and these were all in the RBL group: of the 32 participants that did not receive the procedure, 24 participants were in the HAL group compared with 8 in the RBL group.

**Table 3:** Reason for withdrawal (reasons for withdrawal from treatment are indicated under “Prior to treatment”)

Reason for withdrawal	HAL *N* = 24	RBL *N* = 11
Prior to treatment		
Found to be ineligible after randomisation	0	2
Participant withdrew consent	15	3
Lost to follow up prior to procedure	6	2
Symptoms resolved/treated elsewhere	2	1
Ineligible at time of procedure	1	0
After treatment		
Participant withdrew consent	0	3

Reproduced with permission from Brown et al. 2016 [27]. *HAL* haemorrhoidal artery ligation, *RBL* rubber band ligation

**Fig. 2:**
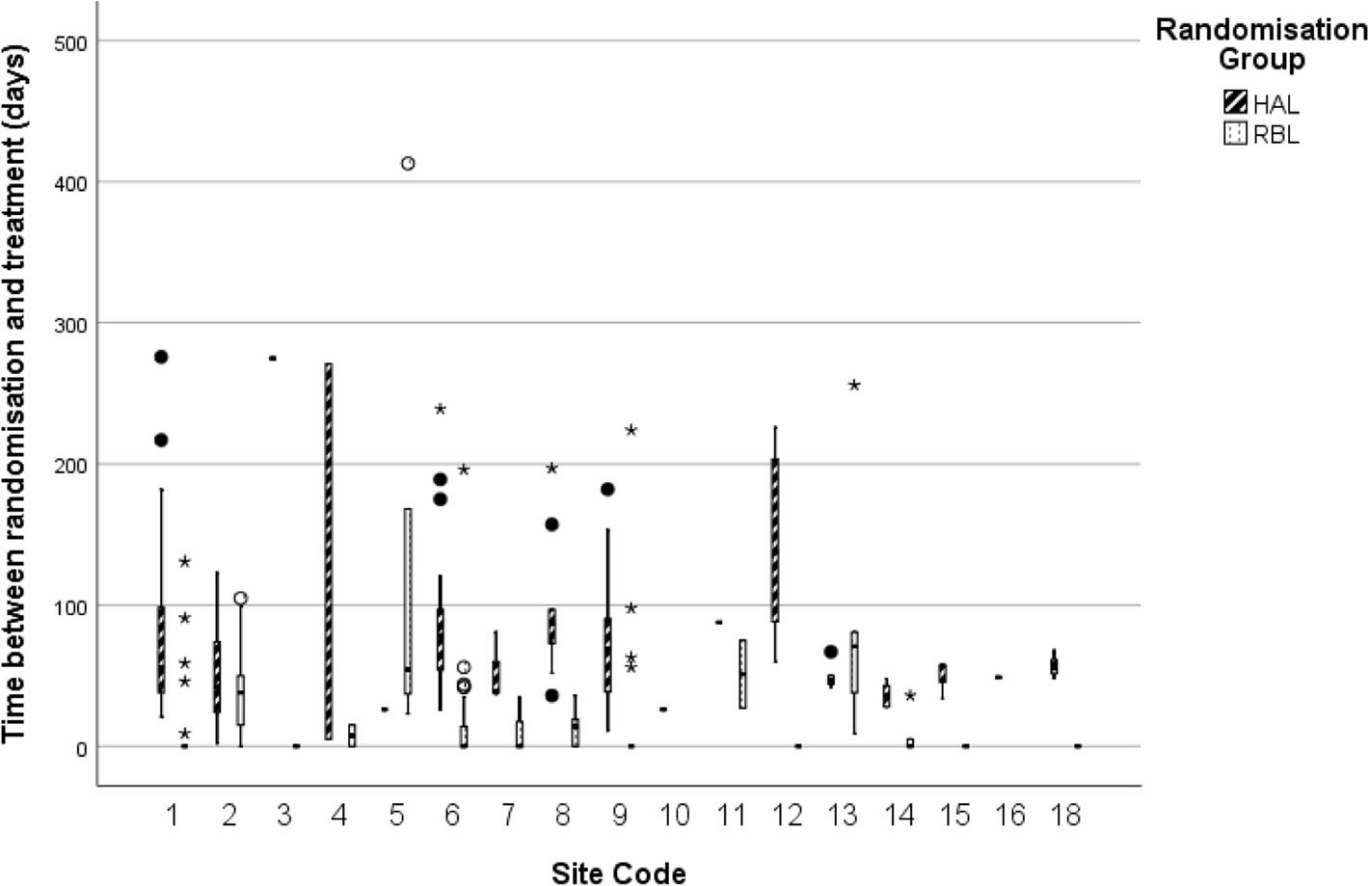
Time to procedure by site and treatment arm (days). HAL, haemorrhoidal artery ligation; RBL, rubber band ligation

Figure 2 shows the time between randomisation and treatment for participants at each site, excluding site 17, which randomised no participants. The median waiting times were longer for HAL (62 days) than that for RBL (0 days) as RBL was often done on the day of randomisation at the sites.

**Fig. 3:**
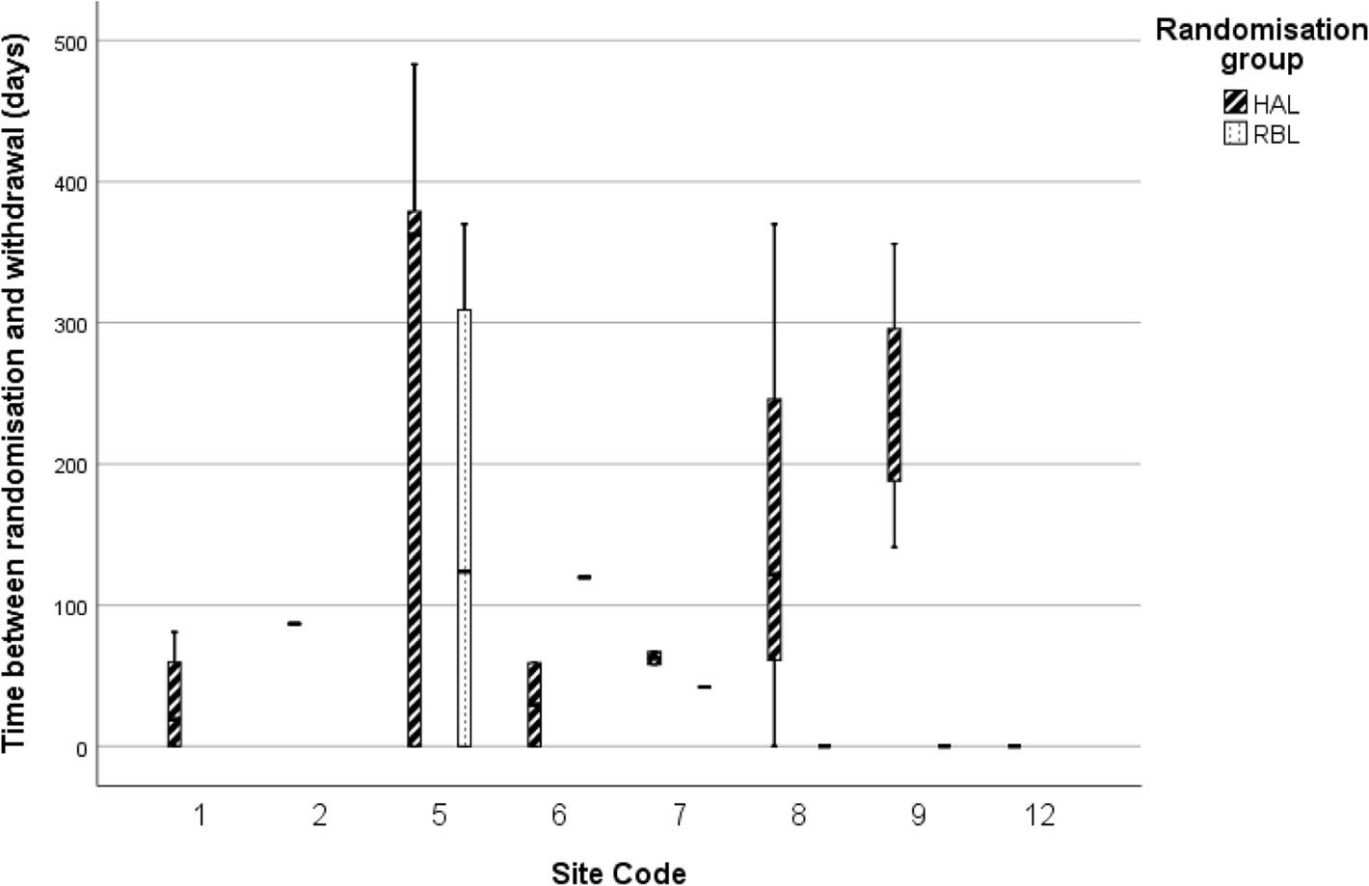
Time to withdrawal (prior to treatment) by site and treatment arm (days). Figure includes only those sites experiencing participant attrition prior to treatment. HAL, haemorrhoidal artery ligation; RBL, rubber band ligation

Figure 3 shows that withdrawal prior to treatment in the HAL group occurred after waiting longer than participants who withdrew in the RBL group. Withdrawal of consent often occurred when contacting patients to book them in for treatment or discuss their waiting time. The majority of participants who withdrew prior to treatment did so after waiting more than a month for the procedure (29/32). Site 5 had particular problems with their waiting times, eventually stopping non-urgent surgical procedures: eight participants did not receive the HAL procedure, and four did not receive the RBL procedure due to withdrawal of consent, loss to follow up, or receiving treatment elsewhere.

### Changes in symptoms between randomisation and procedure

**Fig. 4:**
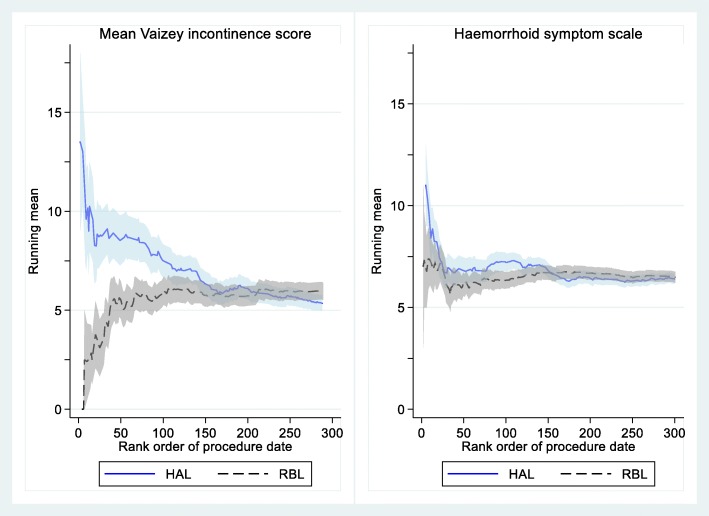
Baseline patient-reported haemorrhoid symptom score and incontinence as taken on day of procedure. HAL, haemorrhoidal artery ligation; RBL, rubber band ligation

Due to the differences in waiting time between randomisation and procedure (Fig. 2), data from the baseline assessment were reviewed to see if expectation bias or clinical deterioration was evident. The early accumulating data indicated that this was possible. Figure 4 depicts the pre-treatment means for self-reported symptoms and incontinence against time during the recruitment period and, as can be seen, the mean incontinence scores were initially higher in the surgery arm than in the RBL arm; a similar but less pronounced pattern was also noted for symptoms. To address this, a pre-randomisation questionnaire was introduced, with a second questionnaire given on the day of the procedure only where more than a week had elapsed between randomisation and the procedure. The group means converged by the end of recruitment, suggesting the initial differences were artefacts of relatively small sample sizes.

The differences in means between pre-randomisation and pre-treatment (baseline) measures (Table 4) were not significant in any of the patient-reported measures, which reassured us that there was no systematic change due to expectation bias or clinical deterioration. Nevertheless, there were some considerable differences between the two measures on an individual level. To put this into context, the 95% reference intervals for change between randomisation and procedure included 0.5 standard deviations, a magnitude comparable to or exceeding the minimally clinically important difference in many RCTs. Moreover, the variability of the change (the ratio of variances, Table 4) was greater in the HAL arm for two of the four questionnaires (Vaizey faecal incontinence and the Euroqol - 5 dimensions - 5 levels (EQ-5D-5L) questionnaires), suggesting these were either sensitive to temporal trends and/or lacked test-retest validity - although on average these changes cancelled each other out in terms of the mean change.

**Table 4:** Agreement between self-completed measures of symptoms, incontinence, EQ-5D-5 L, and pain pre-randomisation and pre-treatment (baseline)

Measure	Mean change (95% agreement limits)		Difference in mean change	Ratio of variances
	HAL	RBL		
Haemorrhoid symptom score	0.0 (−3.0, 3.0)	0.1 (−3.0, 3.1)	−0.1 (*p* = 0.823)	0. 96 (*p* = 0.864)
EQ-5D-5L	−0.01 (− 0.13, 0.11)	−0.00 (− 0.12, 0.11)	−0.01 (*p* = 0.508)	1.11 (*p* = 0.691)
Vaizey Faecal incontinence score	−0.1 (−5.6, 5.3)	0.4 (−2.7, 3.5)	−0.5 (*p* = 0.231)	3.14 (p < 0.001)
VAS pain	0.2 (− 3.8, 4.2)	−0.0 (− 2.5, 2.4)	0.2 (*p* = 0.479)	2.63 (*p* < 0.001)

Reproduced with permission from Brown et al. 2016 [27]. *HAL* haemorrhoidal artery ligation, *RBL* rubber band ligation, *EQ-5D-5L* Euroqol - 5 dimensions - 5 levels questionnaire, *VAS* visual analogue scale

### Primary outcome

Our primary outcome was recurrence at 1 year post-treatment. This included a patient-reported outcome measure supplemented by a case note review of further treatment and haemorrhoid-related events.

**Fig. 5:**
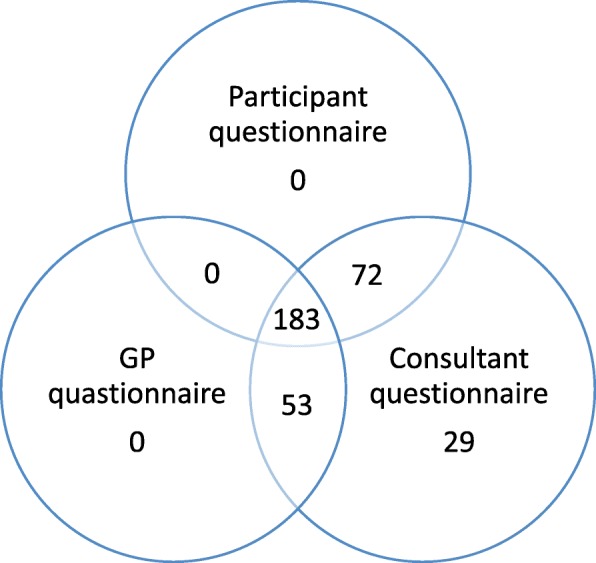
Source of primary outcome data collection. GP, general practitioner

Figure 5 and Table 5 show that data were collected from all three sources (patient, consultant, and GP) on 183 participants and the best method for data collection was from the hospital notes (consultant questionnaire), with 337 (96% of the sample of 350) of these completed. If we had only relied on the patient-reported outcome we would have had outcome data on 255 participants (73% of the sample of 350). Figure 5 shows that recurrence was reported by 71 participants at 1 year but that 83 participants had received further treatment as reported in the GP or consultant questionnaire. In total 135 participants were found to have had a recurrence, which would have been underestimated had only one of these sources been used for the primary outcome.

**Table 5:** Data sources for recurrence at one year

	RBL (*N* = 176)	HAL (*N* = 161)
Recurrence at one year (total)	87 (49%)	48 (30%)
Self-reported recurrence^a^	37 (29%^b^)	34 (29%^b^)
Data from GP and consultant questionnaires^a^	60 (35%)	23 (14%)

Adapted with permission from Brown et al. 2016 [27]. *HAL* haemorrhoidal artery ligation, *RBL* rubber band ligation, *GP* general practitioner

^a^Individuals may contribute data to both measures of recurrence

^b^Denominator is number of patients returning questionnaire

### Consolidated standards of reporting trials (CONSORT) diagram

**Fig. 6:**
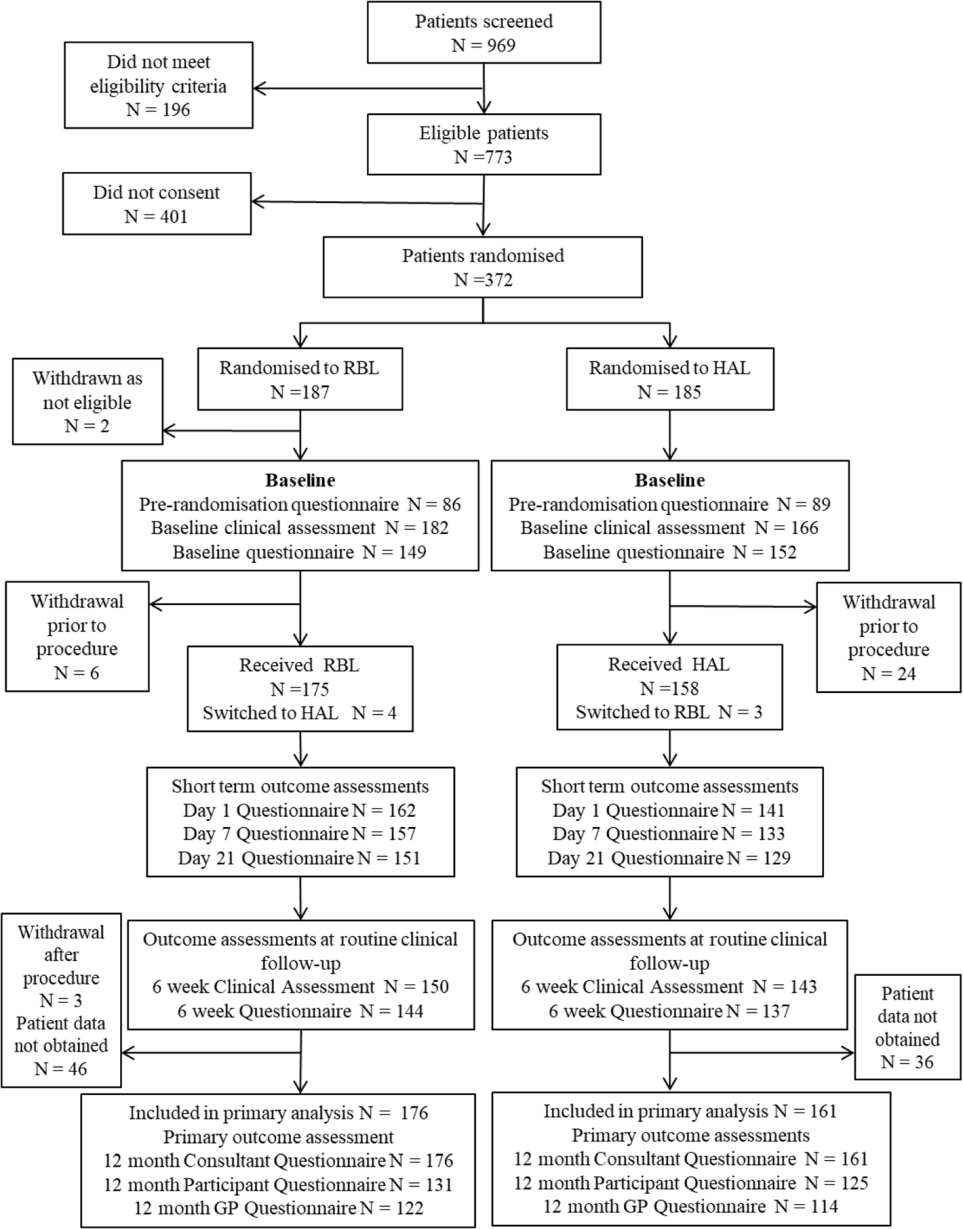
Participant flow diagram. **Reproduced with permission from Brown et al. 2016** [27]. **HAL, haemorrhoidal artery ligation; RBL, rubber band ligation, GP, general practitioner**

The complete trial information in relation to recruitment and data collection is provided in the CONSORT diagram in Fig. 6. Overall there was a good rate of recruitment, with 372 out of the 969 screened recruited and a low rate of attrition, with 337 (90.6%) contributing to the primary outcome. As our original target was 350, our attrition rate was 3.7%, less than the 5% used for our sample size calculation [26, 27].

## Discussion

### Statement of findings

The HubBLe trial is a relatively rare example of a surgical trial that recruited to target and maintained adequate participant follow up. The HubBLe team reduced the risk of project failure by addressing four key areas. We increased the chances of recruiting to target with broad eligibility criteria as suggested elsewhere [5, 33–36]; forecasting recruitment rates based on previous studies [34, 37, 60]; accounting for screen failures in resourcing recruitment activity; and highlighting the issue of equipoise in the training of recruitment staff as proposed by Donovan and colleagues [13–15]. However, as expected for treatments of differing intensity [11], patient preference for treatment was still a barrier to recruitment. In addition, we reduced the risk of delays to recruitment, as recommended [42–46], by having early discussions with sites to secure funding for recruitment activity. As waiting times have been shown to be a barrier to treatment [21, 61], we anticipated differences in the time from randomisation to treatment in each arm [54], reducing the risk of bias due to differential attrition. We avoided variation in length of follow up between arms by collecting baseline data on the day of surgery in addition to randomisation. We then compared the data to check for the influence of expectation bias on self-report measures, as suggested by Schulz [24], but found no systematic differences between the two timepoints. In “Primary outcome” we reduced the risk of unreliable data by triangulating across three sources [58].

### Strengths and limitations

This paper shows how a trial can use a battery of evidence-based methods and the collected experience of a clinical trials unit [62] to achieve study objectives. We were not resourced to conduct qualitative research alongside the trial to understand and address recruitment issues, as is now best practice [19, 63] and our approach was somewhat ad hoc, with systematic evaluation of the strategies not conducted. For instance, formal feedback on the recruitment training video, which was produced without any funding, was not elicited to improve future efforts and screening data may have been incomplete, as is common in RCTs [64], and time spent screening patients was not monitored so we cannot determine if our costing of recruitment activity was appropriate.

### Meaning and application of findings

The data in this paper, such as those on consent rates and attrition prior to treatment, can be added to the reference class for surgical trials and used in future forecasting. Data showing the imbalance in recruitment between trial sites are important: difference in site capability is frequently observed and has implications for trial planning [65–69], especially in allowing over-recruitment in site contracts to compensate for less able sites.

The paper highlights issues around waiting times for surgery in the UK [25, 70–73] and how this can differ between arms in type 3 trials [9], which should be accounted for in the sample size estimation and when deciding on the timing of data collection. Consideration needs to be given to whether baseline data should be collected at randomisation or on the day of treatment [25]; though our data show there is little difference between timepoints. Decisions on whether follow-up data should be anchored to randomisation or to the trial treatment also need to be made: if HubBLe had anchored follow up to randomization rather than to trial treatment, the time between treatment and follow up would have been greater in the RBL group due to the longer waiting times for HAL, which in turn could have affected the primary outcome of recurrence.

### Unanswered questions and future research

It may not be possible to repeat the comparatively generous allocation of service support costs to this trial in the UK due to the subsequent introduction of the Department of Health’s new costing template (the Activity Capture and Attribution Tool, or ACAT) and the UK Clinical Research Facility Network Intensity Tool to cost research nurse activity. Our experience on more recent trials is that these two tools may considerably underestimate the research nurse time necessary to undertake essential research procedures, threatening the success of the recruitment effort and the integrity of research data. Published workload models estimating staff resource for RCTs [74–79] are often criticised for over-simplicity, and their use can lead to staff burnout and poor implementation [80–82]. The rise of surgical trainee networks in the UK as a force in recruitment may go some way to compensating for the pressures on costs in public sector research, where trainees can be incentivised and co-ordinated to recruit and follow up study participants [83].

Stronger evidence for recruitment and retention strategies in RCTs is required to improve trial efficiency and meet trial objectives. Trial Forge [84] is an initiative set up to address the lack of evidence in trial decision-making, which will go some way to evaluate recruitment and retention strategies that can be used across RCTs. The use of studies within a trial (SWATs) to find evidence for implementation of RCTs is becoming more commonplace and could be used to assess some of the strategies presented in this paper [85–87].

## Conclusions

Recruitment to and retention in trials comparing surgical interventions of different intensity is challenging but achievable. This paper provides a range of evidence-based and experience-based approaches, which collectively resulted in meeting our study objectives and from which lessons may be transferable.

## Data Availability

Requests for further data not available in this publication can be directed at Sheffield Clinical Trials Research Unit. Email: ctru@sheffield.ac.uk Tel: 0114 222 0866.

## Abbreviations

ACAT: Activity Capture And Attribution Tool
CCG: Clinical Commissioning Group
DMEC: Data Monitoring and Ethics Committee
EQ-5D-5L: Euroqol - 5 dimensions - 5 levels questionnaire
GP: General pracitioner
HA: Haemorrhoidal artery ligation
HTA: Health Technology Assessment
HRQoL: Health-related quality of life
HubBLe: Haemorrhoidal artery ligation versus rubber band ligation for haemorrhoids trial
LRN: Local Research Network
NHS: National Health Service
NIHR: National Institute for Health Research
R&D: Research and development
RBL: Rubber band ligation
RCT: Randomised controlled trial
SWAT: Studies within a trial
WTE: Whole time equivalent
